# Supplementary material for: Administration of Enfortumab Vedotin after Immune-Checkpoint Inhibitor and the Prognosis in Japanese Metastatic Urothelial Carcinoma: A Large Database Study on Enfortumab Vedotin in Metastatic Urothelial Carcinoma
Source: Cancers (Basel). 2023 Aug 23;15(17):4227. doi: 10.3390/cancers15174227 (PMC10486515; doi:10.3390/cancers15174227)
Supplement: Supplementary file 1 [file cancers-15-04227-s001.zip › cancers-2554335-supplementary.pdf]

Table S1. Multivariable analyses for cancer specific survival

Variables	HR	95%CI		p value
		Lower	Upper	
Age 70yrs over	0.83	0.60	1.14	0.241
Male	0.95	0.65	1.40	0.801
Bladder Cancer	0.77	0.55	1.09	0.143
EV induction	0.82	0.58	1.16	0.262
Cystecyomy	1.19	0.74	1.92	0.466

HR: hazard ratio, EV: enfortumab vedotin

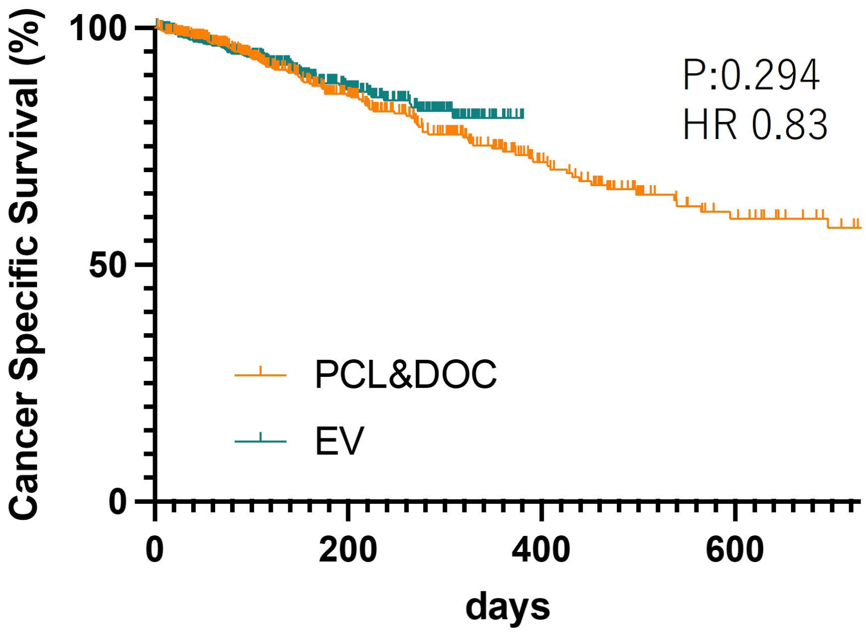

	0	100	200	300	400	500	600
PCL/DOC	443	325	222	146	93	59	43
EV	563	360	192	67	0	0	0

EV: Enfortumab vedotin, DOC: docetaxel, PCL: paclitaxel

Figure S1. Kaplan Meier Curve for cancer specific survival: paclitaxel/docetaxel vs. enfortumab vedotin.
